# Disentangling clinical heterogeneity in schizophrenia through neuropsychophysiological profiles

**DOI:** 10.1192/j.eurpsy.2025.420

**Published:** 2025-08-26

**Authors:** G. Agostoni, S. Zago, M. Bechi, J. Sapienza, R. Cavallaro, V. Bambini, G. Arcara, M. Bosia

**Affiliations:** 1 Vita-Salute San Raffaele University, Milan; 2 IRCCS San Camillo, Venezia; 3 IRCCS San Raffaele, Milan; 4 Istituto Universitario di Studi Superiori IUSS, Pavia, Italy

## Abstract

**Introduction:**

Cognitive disruption is a key feature in schizophrenia, and the identification of the neurophysiological underpinnings is of particular interest. One of the most promising markers of cognition is aperiodic activity, which is considered as a proxy measure of excitation inhibition (E/I) balance, stemming from the equilibrium between glutamatergic and GABAergic neurotransmission. E/I alteration has been found in multiple disorders, but its relationship with cognition in schizophrenia has never been explored.

**Objectives:**

This study aims at demonstrating the link between aperiodic activity and cognition in schizophrenia, and at creating neuropsychophysiological profiles, associated with clinical and functional features.

**Methods:**

48 patients with schizophrenia were assessed for cognition, well-being and the severity of psychopathology and underwent an electroencephalogram (EEG) recording during a resting state. EEG tracks were processed to extract aperiodic parameters (offset and exponent). Pearson correlation analyses between aperiodic and cognitive measures were performed. Aperiodic indexes and the related cognitive domains were used to create neuropsychophysiological profiles, using a two-step cluster analysis. Analyses of Variance were performed to characterize significant differences in severity of psychopathology and well-being between profiles. Moderation analyses were run to identify the interplay between profiles, psychopathological severity and well-being.

**Results:**

The mean aperiodic offset was -12.45 (± 0.65), while the mean exponent was 0.85 (± 0.28). Significant correlations with aperiodic parameters were found for: Working Memory, Processing Speed and Psychomotor Speed (Fig1-2). Cluster analysis identified two profiles (Profile1 N=15, Profile2 N=33): Profile1 had a higher offset, a steeper slope. ANOVAs revealed that Profile1 showed significantly higher scores in Working Memory and Processing speed, and lower levels of General Psychopathology, Anxiety/Depression and Uncontrolled Excitement and Hostility. Lastly, mediation models, showed an interaction between Profiles (R^2^=0.21, p=.04) and Anxiety/Depression as well as between Profiles and General psychopathology (R^2^=0.30, p=.003) on Self-Acceptance, with significant negative relationship only in Profile2 in both models (Fig 3).

**Image 1:**

**Figure:**
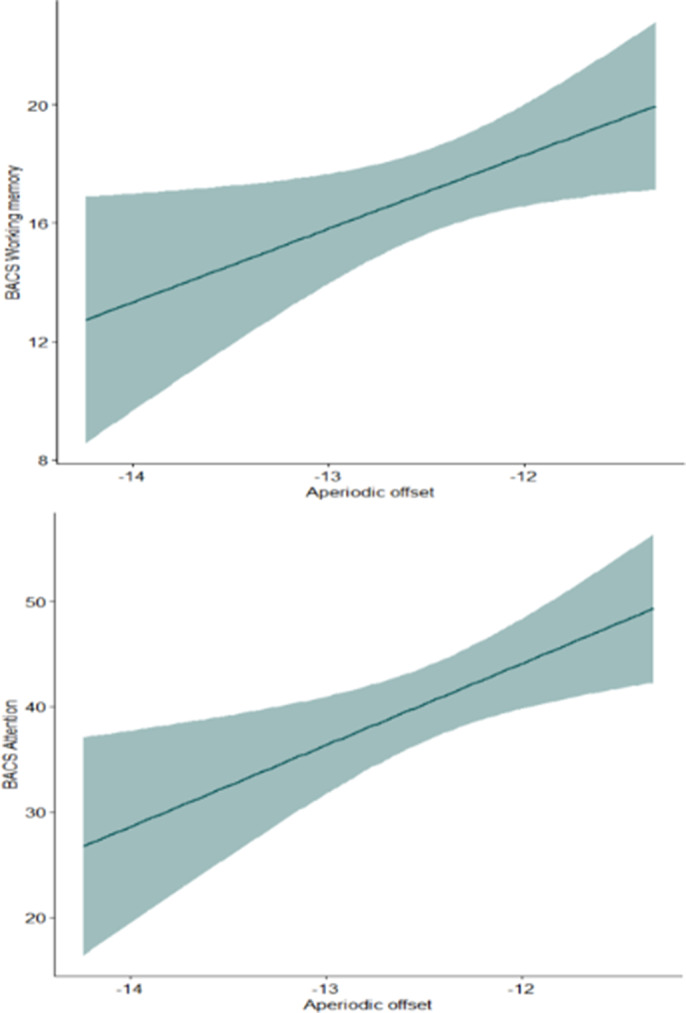

**Image 2:**

**Figure:**
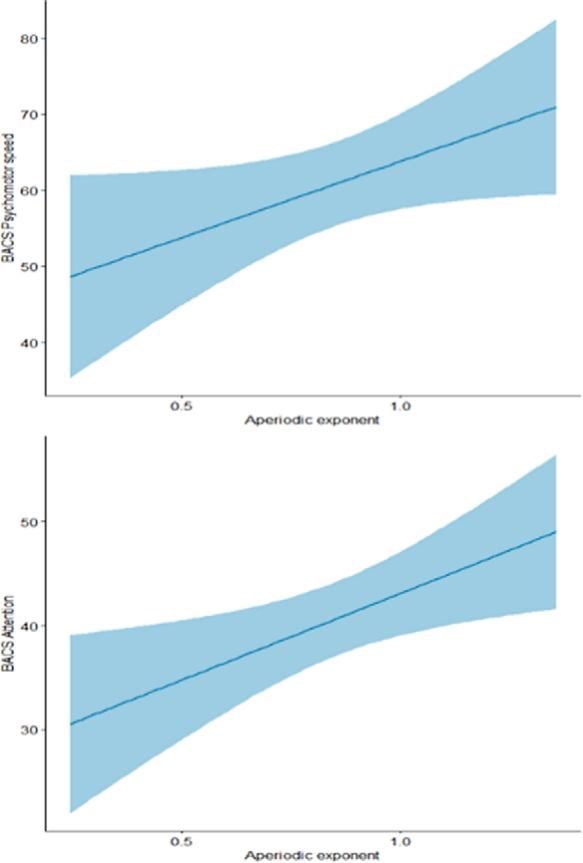

**Image 3:**

**Figure:**
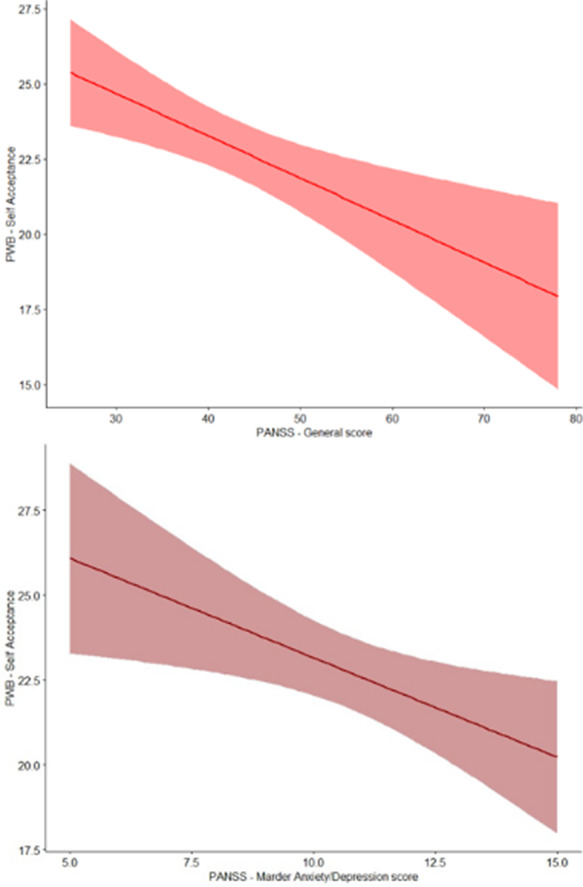

**Conclusions:**

Our data support an altered E/I balance in schizophrenia and innovatively show a direct link between aperiodic activity and the cognitive disruption. This relationship is further confirmed by the identification of two profiles, characterized by distinct neuropsychological and neurophysiological measures, with a flatter aperiodic slope, corresponding to an altered E/I balance, being associated with more severe cognitive impairment and illness severity. The clinical relevance is highlighted by the interplay between symptoms severity and neuropsychophysiological patterns on subjective well-being.

**Disclosure of Interest:**

None Declared

